# Incidence and prognosis of intra-abdominal hypertension in critically ill medical patients: a prospective epidemiological study

**DOI:** 10.1186/2110-5820-2-S1-S3

**Published:** 2012-07-05

**Authors:** Patricia Santa-Teresa, Javier Muñoz, Ignacio Montero, María Zurita, María Tomey, Luis Álvarez-Sala, Pablo García

**Affiliations:** 1 Intensive Care Unit, Hospital General Universitario Gregorio Marañón, Calle Doctor Esquerdo 46, Madrid, 28007, Spain; 2 Department of Internal Medicine, Hospital General Universitario Gregorio Marañón, Calle Doctor Esquerdo 46, Madrid, 28007, Spain

**Keywords:** intra-abdominal hypertension, abdominal compartment syndrome, intra-abdominal pressure, multiple organ failure, critically ill patients, intensive care.

## Abstract

**Introduction:**

The aim of this study was to determine the incidence of intra-abdominal hypertension (IAH) in patients with two or more categorized risk factors (CRF) for IAH, and their morbidity and mortality during their intensive care unit (ICU) stay.

**Methods:**

Prospective cohort study carried out at a medical ICU. A total of 151 medical patients were enrolled during a period of 3 months. After ICU whole staff training, we conducted daily screening of the four CRF for IAH based on the World Society of Abdominal Compartment Syndrome (WSACS) guidelines (namely, diminished abdominal wall compliance, increased intraluminal content, increased abdominal content, and capillary leak syndrome or fluid resuscitation). In those patients with risk factors of at least two different categories (≥2 CRF), intra-abdominal pressure (IAP) was measured every 8 h during ICU stay. Data included demographics, main diagnosis on admission, severity scores, cumulative fluid balance, daily mean IAP, resolution of IAH, days of ICU and hospital stay, and mortality.

**Results:**

Eighty-seven patients (57.6%) had ≥2 CRF for IAH, 59 (67.8%) out of whom developed IAH. Patients with ≥2 CRF had a significantly higher mortality rate (41.4 vs. 14.3%, *p *< 0.001). Patients with IAH had higher body mass index, severity scores, organ dysfunctions/failures, number of CRF for IAH, days of ICU/hospital stay and hospital mortality rate (45.8 vs. 32.1%, *p *= 0.22). Non-resolution of IAH was associated with a higher mortality rate (64.7 vs. 35.3%, *p *= 0.001). None of the cohort patients developed abdominal compartment syndrome. The multivariate analysis showed that IAH development (odds ratio (OR) 4.09; 95% confidence interval (CI) 0.83-20.12) was a non-independent risk factor for mortality, and its non-resolution (OR 13.15; 95% CI 22.13-81.92) was an independent risk factor for mortality.

**Conclusions:**

Critically ill medical patients admitted to ICU with ≥2 CRF have high morbidity, mortality rate, and incidence of IAH, so IAP should be measured and monitored as recommended by the WSACS. Our study highlights the importance of implementing screening and assessment protocols for an early diagnosis of IAH.

## Introduction

Intra-abdominal hypertension (IAH) is associated with adverse outcomes in critically ill patients [1,2]. Elevated intra-abdominal pressure (IAP) above physiologic limits has deleterious effects on end-organ function due to pathophysiologic changes such as a drop in cardiac output, diminished chest wall compliance, decreased renal and visceral blood flow, and increased intracranial pressure [3-11].

IAH has traditionally been associated with patients undergoing abdominal surgery and trauma patients who require aggressive volume resuscitation [12-16]. Several studies have demonstrated that IAH is also frequently present in critically ill nonsurgical patients, and is now considered to be associated with the general process of inflammation and resuscitation [17-22]. However, establishing comparisons for incidence, morbidity and mortality rates in medical patients from data of published clinical trials is difficult, mainly due to three reasons: firstly, the absence of uniform definitions before consensus guidelines, which allow us to reproduce and interpret the results; secondly, the different methods used for IAP measurement; and lastly, the fact that most clinical studies have been performed on mixed populations (both medical and surgical).

The World Society of Abdominal Compartment Syndrome (WSACS) has published a consensus statement including definitions and recommendations for the screening and management of IAH and abdominal compartment syndrome (ACS) [23,24]. Following this consensus, the aim of this study was to determine the incidence of IAH in exclusively critically ill medical patients with high risk of IAH development, and their morbidity and mortality during their intensive care unit (ICU) stay.

## Materials and methods

This study was conducted in a university hospital medical ICU in accordance with a protocol that was approved by the local ethics committee. The study fulfilled the requirements of the Declaration of Helsinki, and included an informed consent from the patients on admission (or their family if necessary) to participate and publish the results.

All medical patients admitted to the ICU during a three-month period and expected to stay > 24 h were prospectively enrolled, provided they needed a bladder catheter. Exclusion criteria were ICU stay < 24 h, age < 18 years, pregnancy, contraindication for intravesical pressure measurement (pelvic fracture, hematuria, or neurogenic bladder), and bladder surgery. WSACS consensus for assessment of IAH was implemented as a new protocol [23,24].

### Data collection

#### Demographic data

Age, gender, body mass index (BMI) expressed as kg/m^2 ^[25], main diagnosis on admission, cumulative fluid balance, length of ICU and hospital stay, and hospital mortality were considered in collecting the patients' data. Patients were followed until death or hospital discharge, whatever came first.

#### Organ dysfunction

On admission, APACHE II (Acute Physiology and Chronic Health Evaluation) and SOFA (Sequential Organ Failure Assessment) scores were recorded using the worst values of the day [26,27]. Organ dysfunction/failure was evaluated using the SOFA score (dysfunction = subscore ≤2, failure = subscore ≥3). The SOFA maximum score was based on the worst value of each organ system during the ICU stay.

#### Risk factors for IAH

All patients were prospectively screened for risk factors (RF) of IAH on admission and daily during their ICU stay until death or discharge, based on four categories from the WSACS algorithm (Table 1). Those patients who presented any RF of at least two different categories (≥2 categorized risk factors (CRF)), at any time on admission or during the ICU stay, were considered to have a high risk of developing IAH, and IAP was monitored (starting the day they were found to have ≥2 CRF). Those patients with < 2 CRF on admission and daily during the ICU stay were considered to have a low risk of developing IAH according to WSACS recommendations and their IAP measurement was not recorded. For the main analysis, we calculated the number of RF as categories (one minimum, four maximum) on admission (first 24 h) and during the ICU stay (mean RF categories present per day).

**Table 1:** Categorized risk factors for intra-abdominal hypertension

Diminished abdominal wall compliance
• Mechanical ventilation
• Abdominal surgery with primary fascial or tight closure
• Major trauma
• Major burns
• Prone positioning
• Head of bed > 30 degrees
• Body mass index ≥ 30 kg/m^2 ^or morbid obesity
Increased intra-luminal contents
• Gastroparesis (gastric dilation or gastric residual > 500 mL).
• Ileus, paralytic or mechanical (abdominal distention or absence of bowel sounds)
• Colonic pseudo-obstruction
Increased abdominal contents
• Hemoperitoneum or pneumoperitoneum
• Ascites secondary to liver dysfunction
• Ascites secondary to liver dysfunction
• Other intra-abdominal injuries (peritonitis, abscess)
Capillary leak syndrome or fluid resuscitation
• Acidosis (arterial pH < 7.2)
• Hypotension (systolic blood pressure < 90 mmHg or mean arterial pressure < 70 mmHg or a systolic blood pressure decrease > 40 mmHg or > 2 standard deviation below normal for age in the absence of other causes of hypotension)
• Hypothermia (core temperature < 33°C).
• Multiple transfusions (> 10 units of blood)
• Coagulopathy (platelets < 55,000/mm^3 ^or prothrombin time < 15 s or partial thromboplastin time > 2 times normal or international standardized ratio > 1.5)
• Massive fluid resuscitation (> 5 L of colloid or crystalloid)
• Acute pancreatitis
• Oliguria (urine output < 500 mL).
• Sepsis (American-European Consensus Conference definitions)
• Major trauma
• Major burns
• Damage control laparotomy

#### IAP measurement

IAP was measured intravesically using a closed-system Foley bladder catheter according to the modified Kron technique [28,29] with an instillation volume of 25 ml of saline. Abdominal perfusion pressure (APP) was calculated as mean arterial pressure minus IAP [30,31]. Measurements of IAP and APP were recorded every 8 h in patients with ≥2 CRF until resolution of IAH (in case it developed), death, or discharge from the ICU. IAH was defined as a repeated (at least three consecutive values separated by 8 h) elevation of IAP_min _(the lowest value of each of the three IAP measures) ≥12 mmHg [23]. For the main analysis, IAP_mean _and APP_mean _(mean of the three daily values) were calculated per day.

#### IAH characteristics

We recorded the day of onset, days of sustained IAH, surgical abdominal decompression, and resolution. Resolution of IAH was defined as a sustained IAP_min _< 12 mmHg in all measurements recorded every 8 h for a minimum of 48 h; and non-resolution was defined as a sustained IAP_min _≥ 12 mmHg in all measurements until death or discharge from the ICU. IAH was classified according to the time of onset (on admission or during ICU stay) and the development of symptoms (acute, over a period of hours; subacute, over a period of days; or chronic, over a period of months/years). We also recorded the grade of maximal IAP reached during the ICU stay (I, 12-15; II, 16-20; III, 21-25; IV, ≥25 mmHg) [23].

### Statistical analysis

#### Univariate analysis

Continuous non-normally distributed variables were expressed as the median and interquartile range and compared using the nonparametric Mann-Whitney test. Continuous normally distributed variables were expressed as mean ± standard deviation, and compared using the student's *t *test. Categorical variables were expressed as numbers and percentages and compared using the chi-square test or Fisher exact test. All *p *values were two-tailed, and *p *< .05 was considered as statistically significant.

#### Multivariate analysis

The prognostic relevance of two different parameters was analyzed: variables associated with development of IAH and the prognostic value of IAH and its non-resolution. Backward multiple logistic models were used, including all the variables yielding *p *< .2 by univariate analysis and those considered clinically relevant. Possible interactions were tested. The results were summarized as odds ratios their respective 95% confidence intervals. The area under the receiver operating characteristic (ROC) curve was used to assess discrimination; an area under the ROC curve of 1.0 denotes perfect discrimination, whereas a value close to 0.50 indicates no apparent accuracy. The Hosmer-Lemeshow goodness-of-fit test was used to evaluate agreement between the observed and expected results across all the probability strata of the outcome of interest (calibration); *p *> .05 indicates a good fit for the model. Kaplan-Meier curves for survival at 28-day hospital stay were constructed for subgroups with IAH.

## Results

### Criteria for IAP monitoring and incidence of IAH (Figures 1 and 2)

During the study period, 204 patients were admitted. Fifty-three were excluded: 5 refused to participate, 22 had ICU stay < 24 hours, 12 had contraindications for intravesical pressure measurement or bladder surgery, 11 did not require urinary catheterization, 2 were < 18 years and 1 was pregnant. We finally enrolled 151 medical patients, and the entire protocol was completed in all of them. Main diagnosis on admission was cardiopulmonary disease (Table 2). Sixty-four patients presented < 2 CRF (42.4%) on admission and during all the ICU stay, and IAP was not measured; the remaining 87 (57.6%) patients presented ≥2 CRF (high risk group) for IAH (88.5% on admission and 11.5% during ICU stay), and IAP was monitored following the study protocol. In the high risk group, 59 of the 87 patients developed IAH (67.8%): 35 on admission (59.3%) and 24 during the ICU stay (40.7%). Overall 67.8% of the high risk group subjects developed IAH, 40.2% on admission, and another 27.6% during the ICU stay. Figure 2 shows the differences in the evolution of the number of CRF during the ICU stay across different subgroups of patients. Comparisons between survivors and non-survivors in the study cohort and high risk group are shown in Tables 3 and 4. There were no statistical differences in IAP levels between survivors and non-survivors (11.8 vs. 12.0 mmHg, *p *= 0.78).

**Table 2:** Main diagnosis of the study cohort on admission

	Study cohort (*n *= 151)
Cardiopulmonary diagnosis	67 (44.4)
Acute/chronic liver failure	7 (4.6)
Acute pancreatitis	5 (3.3)
Gastrointestinal disease	8 (5.3)
Other intra-abdominal processes	4 (2.6)
Acute renal failure	13 (8.6)
Cardiac arrest	5 (3.3)
Neurologic disease	30 (19.9)
Other	12 (8)

Values are *n *(percent).

**Figure 1:**
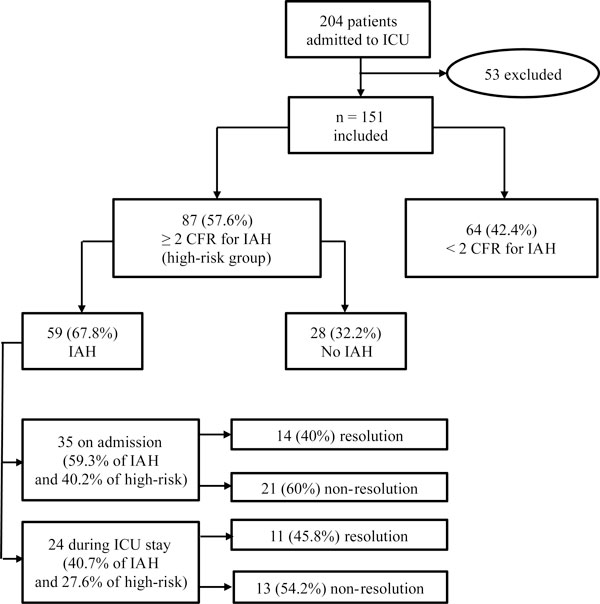
**Algorithm and general results of the study cohort**. IAH, intra-abdominal hypertension; ICU, intensive care unit; CRF, categorized risk factors.

**Figure 2:**
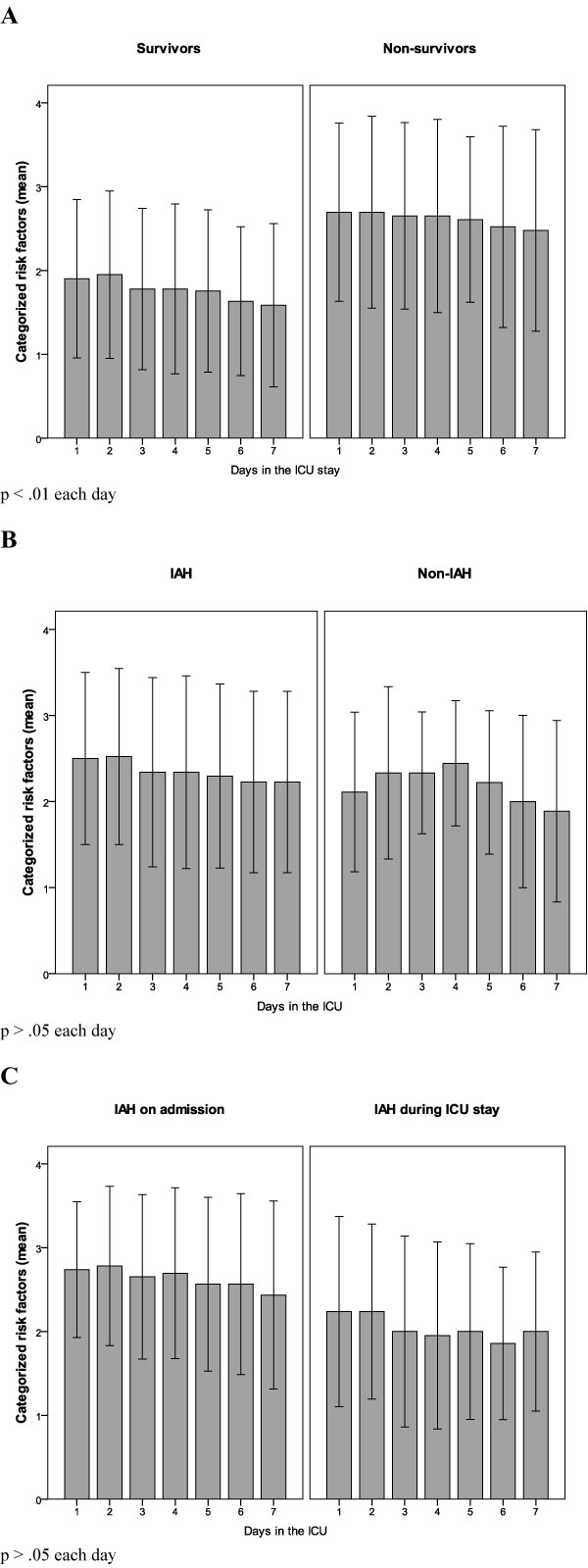
**Differences in the evolution of the number of categorized risk factors during the ICU stay**. Differences in the evolution of the number of categorized risk factors during the ICU stay across survivors and non-survivors in the study cohort (**A**), patients with and without IAH (**B**), and patients with IAH on admission or during the ICU stay (**C**). ICU, intensive care unit; IAH, intra-abdominal hypertension.

**Table 3:** Categorized risk factors between survivors and non-survivors in the study cohort

	Survivors (*n *= 109)	Non-survivors (*n *= 42)	*p *value
Number of categorized risk factors			
On admission	1.5 ± 0.9	2.4 ± 1.1	< .001
During ICU stay	1.6 ± 1	2.8 ± 1.1	< .001
Risk factors during ICU stay			
(*n*, percent)			
Diminished abdominal wall compliance	75 (68.8)	39 (92.8)	= .002
Increased intraluminal contents	20 (18.3)	24 (57.1)	< .001
Increased abdominal contents	10 (9.2)	16 (38.1)	< .001
Capillary leak syndrome/fluid resuscitation	61 (55.9)	36 (85.7)	= .001

ICU, intensive care unit. Data are presented as mean ± standard deviation, unless otherwise indicated.

**Table 4:** Categorized risk factors between survivors and non-survivors in the high risk group

	Survivors (*n *= 51)	Non-survivors (*n *= 36)	*p *value
Number of categorized risk factors			
On admission	2.2 ± 0.8	2.7 ± 0.9	< .001
During ICU stay	2.6 ± 0.7	3.1 ± 0.8	< .001
Risk factors during ICU stay (n, percent)			
Diminished abdominal wall compliance	48 (94.1)	35 (97.2)	< .001
Increased intraluminal contents	20 (39.2)	24 (67.6)	< .001
Increased abdominal contents	10 (19.6)	16 (44.4)	< .001
Capillary leak syndrome/fluid resuscitation	45 (88.2)	34 (94.4)	< .001

Categorized risk factors between survivors and non-survivors in the high risk group (87 patients with ≥2 categorized risk factors). ICU, intensive care unit. Data are presented as mean ± standard deviation, unless otherwise indicated.

### General characteristics for the comparison between high risk and low risk groups (Table 5)

We compared patients with ≥2 CRF (*n *= 87) with those with < 2 CRF for IAH (*n *= 64). Severity of illness, ICU and hospital length of stay, and mortality rate (41.4 vs. 14.3%, *p *< 0.001) were significantly higher in the high risk group.

**Table 5:** General characteristics of the study cohort, and comparisons between patients

	Study cohort	≥2 categorized risk factors	<2 categorized risk factors	*p *value
Patients (*n*, percent)	151 (100)	87 (57.6)	64 (42.4)	-
Age (years)	56.7 ± 17.5	55.8 ± 17.2	58 ± 17.9	NS
Male gender (*n*, percent)	106 (70.2)	66 (75.9)	40 (62.5)	NS
Body mass index (kilograms per square meter)	25.9 ± 5.3	26.3 ± 5.6	25.3 ± 4.9	NS
Acute Physiology and Chronic Health Evaluation score, points	20.1 ± 8.4	22.8 ± 7.9	16.5 ± 7.8	< .001
Sequential Organ Failure Assessment score on day 1, points	6.4 ± 3.9	7.9 ± 3.9	4.4 ± 2.7	< .001
Number of organ failures on day 1, points	3 ± 1.3	3.2 ± 1.3	2.2 ± 1.1	< .001
Sequential Organ Failure Assessment score maximum, points	8.1 ± 5.2	10.5 ± 5.1	4.8 ± 3.1	< .001
Number of organ failures maximum, points	3 ± 1.5	3.8 ± 1.5	2.3 ± 1.1	< .001
Positive cumulative fluid balance during ICU stay	59 (39.1)	34 (39)	25 (39)	NS
ICU stay (days)^a^	5 (3-12)	9 (4-25)	3 (2-4)	< .001
Hospital stay (days)^a^	16 (9-37)	24 (4-55)	13 (8-21)	= .001
Hospital mortality (*n*, percent)	42 (27.8)	36 (41.4)	6 (14.3)	< .001
Number of categorized risk factors				
On admission	1.7 ± 1.0	2.4 ± 0.9	0.8 ± 0.4	< .001
During ICU stay	2.0 ± 1.1	2.8 ± 0.8	0.8 ± 0.3	< .001
Risk factors during ICU stay^b ^(*n*, percent)				
Diminished abdominal wall compliance	114 (75.5)	83 (95.4)	31 (48.4)	< .001
Mechanical ventilation	104 (68.9)	77 (88.5)	27 (42.2)	< .001
Head of bed > 30°	135 (89.4)	83 (95.4)	52 (81.3)	= .005
Body mass index ≥30 kg/m^2^	36 (23.8)	26 (29.9)	10 (15.6)	= .04
Increased intraluminal contents	44 (29.1)	44 (50.6)	0 (0)	< .001
Gastroparesis	37 (24.5)	37 (42.5)	0 (0)	< .001
Ileus	21 (13.9)	21 (24.1)	0 (0)	< .001
Increased abdominal contents	26 (17.2)	26 (29.9)	0 (0)	< .001
Ascites/liver dysfunction	14 (9.3)	14 (16.1)	0 (0)	= .001
Ascites of other causes	4 (2.6)	4 (4.6)	0 (0)	NS
Capillary leak syndrome/fluid resuscitation	97 (64.2)	79 (90.8)	18 (28.1)	< .001
Acidosis	32 (21.2)	28 (32.2)	4 (6.3)	< .001
Hypotension	68 (45)	61 (70.1)	7 (10.9)	< .001
Hypothermia	7 (4.6)	7 (8)	0 (0)	= .01
Multiple transfusions	3 (2)	3 (3.4)	0 (0)	NS
Coagulopathy	48 (31.8)	40 (46)	8 (12.5)	< .001
Massive fluid resuscitation	49 (32.5)	40 (46)	9 (14.1)	< .001
Acute pancreatitis	5 (3.3)	5 (5.7)	0 (0)	NS
Oliguria	43 (28.5)	35 (40.2)	8 (12.5)	< .001
Sepsis	68 (45)	56 (64.4)	12 (18.8)	< .001

General characteristics of the study cohort, and comparisons between patients with ≥2 vs. <2 categorized risk factors for intra-abdominal hypertension. ^a^Data are presented as median and interquartile range. ^b^Abdominal surgery with primary fascial or tight closure, major trauma, major burns, prone positioning, colonic pseudo-obstruction, hemoperitoneum or pneumoperitoneum; other intra-abdominal injuries and damage control laparotomy are not included due to the very low number of patients with these risk factors. APACHE II, Acute Physiology and Chronic Health Evaluation; CRF, categorized risk factors; IAH, intra-abdominal hypertension; ICU, intensive care unit; NS, not significant; SOFA, Sequential Organ Failure Assessment. Data are presented as mean ± standard deviation, unless otherwise indicated.

### General characteristics for the comparison between IAH and non-IAH groups (Tables 6 and 7)

We compared patients who developed IAH with ≥2 CRF (*n *= 59) with those who did not despite having ≥2 CRF (*n *= 28). Mortality rate was higher in patients with IAH, although this did not reach statistical (45.8 vs. 32.1%, *p *< 0.22) significance. Patients with IAH were older, had a higher BMI, higher severity scores and proportion of organ failure, and had a longer ICU and hospital stay. Increased abdominal content (39 vs. 10.7%, *p = *0.007) and IAP_mean _(13 + 1 vs. 8 + 1 mmHg, *p *< 0.001) were also significantly higher.

**Table 6:** General characteristics of the patients with ≥2 categorized risk factors and comparisons between patients

	≥2 Categorized risk factors	Intra-abdominal hypertension	No intra-abdominal hypertension	*p *value
Patients (*n*, percent)	87 (100)	59 (67.8)	28 (32.2)	
Age (years)	55.8 ± 17.2	59.3 ± 16.4	48.5 ± 16.6	.006
Male gender (*n*, percent)	66 (75.9)	44 (74.6)	22 (78.6)	NS
Body mass index (kilograms per square meter)	26.3 ± 5.6	27.7 ± 5.6	23.4 ± 4.2	< .001
APACHE II score, points	22.8 ± 7.9	24.4 ± 7.8	19.5 ± 7.2	.007
SOFA score on day 1, points	7.9 ± 3.9	8.8 ± 4.0	6.2 ± 3.3	.004
Number of organ failures on day 1, points	3.2 ± 1.3	3.5 ± 1.3	2.5 ± 1.2	.002
SOFA score maximum, points	10.5 ± 5.1	11.7 ± 4.7	7.9 ± 4.9	.001
Number of organ failures maximum, points	3.8 ± 1.5	4.2 ± 1.4	3.0 ± 1.4	< .001
Positive cumulative fluid balance during ICU stay	34 (39)	25 (42.4)	9 (32.1)	NS
ICU stay (days)^a^	9 (4-25)	11 (7-29)	4 (3-9)	< .001
Hospital stay (days)^a^	24 (4-55)	29 (13-61)	15 (6-34)	.005
Hospital mortality (*n*, percent)	36 (41.4)	27 (45.8)	9 (32.1)	NS
Number of categorized risk factors				
On admission	2.4 ± 0.9	2.5 ± 0.9	2.1 ± 0.8	.05
During ICU stay	2.8 ± 0.8	2.9 ± 0.8	2.4 ± 0.6	.006
Risk factors during ICU stay^b ^(*n*, percent)				
Diminished abdominal wall compliance	83 (95.4)	57 (96.6)	26 (92.9)	NS
Mechanical ventilation	77 (88.5)	51 (86.4)	26 (92.9)	NS
Head of bed > 30°	83 (95.4)	57 (96.6)	26 (92.9)	NS
Body mass index ≥30 kg/m^2^	26 (29.9)	24 (40.7)	2 (7.1)	.001
Increased intraluminal contents	44 (50.6)	33 (55.9)	11 (39.3)	NS
Gastroparesis	37 (42.5)	28 (47.5)	9 (32.1)	NS
Ileus	21 (24.1)	16 (27.1)	5 (17.9)	NS
Increased abdominal contents	26 (29.9)	23 (39)	3 (10.7)	.007
Ascites/liver dysfunction	14 (16.1)	12 (20.3)	2 (7.1)	NS
Ascites of other causes	4 (4.6)	4 (6.8)	0 (0)	NS
Capillary leak syndrome/fluid resuscitation	79 (90.8)	55 (93.2)	24 (85.7)	NS
Acidosis	28 (32.2)	22 (37.3)	6 (21.4)	NS
Hypotension	61 (70.1)	46 (78)	15 (53.6)	.02
Hypothermia	7 (8)	5 (8.5)	2 (7.1)	NS
Multiple transfusions	3 (3.4)	2 (3.4)	1 (3.6)	NS
Coagulopathy	40 (45.9)	30 (50.8)	10 (35.7)	NS
Massive fluid resuscitation	40 (46)	30 (50.8)	10 (35.7)	NS
Acute pancreatitis	5 (5.7)	5 (8.5)	0 (0)	.005
Oliguria	35 (40.2)	27 (45.8)	8 (28.6)	NS
Sepsis	56 (64.4)	40 (67.8)	16 (57.1)	NS
IAP during ICU stay	12 ± 3	13 ± 1	8 ± 1	< .001
APP mean during ICU stay	74 ± 13	73 ± 12	74 ± 15	NS

General characteristics of the patients with ≥2 categorized risk factors, and comparisons between patients with and without intra-abdominal hypertension. ^a^Data are presented as median and interquartile range. ^b^Abdominal surgery with primary fascial or tight closure, major trauma, major burns, prone positioning, colonic pseudo-obstruction, hemoperitoneum or pneumoperitoneum; other intra-abdominal injuries and damage control laparotomy are not included due to the very low number of patients with these risk factors. APACHE II, APP, abdominal perfusion pressure; Acute Physiology and Chronic Health Evaluation; CRF, categorized risk factors; IAH, intra-abdominal hypertension; IAP, intra-abdominal pressure (mmHg); ICU, intensive care unit; NS, not significant; SOFA, Sequential Organ Failure Assessment. Data are presented as mean ± standard deviation, unless otherwise indicated.

**Table 7:** Comparison of organ failure according to SOFA score between IAH and non-IAH patients

	Intra-abdominal hypertension(*n *= 59)	No intra-abdominal hypertension (*n *= 28)	*p *value
Organ failures on day 1			
Respiratory	56 (94.9)	27 (96.5)	NS
Circulatory	43 (72.8)	11 (39.2)	< .003
Renal	32 (54.2)	4 (14.6)	< .001
Hepatic	21 (35.6)	6 (21.4)	NS
Neurological	28 (47.5)	16 (67.1)	NS
Coagulation	28 (47.5)	7 (25)	= .04
Organ failures during ICU stay			
Respiratory	58 (98.4)	26 (92.8%)	NS
Circulatory	47 (79.7)	16 (57.1%)	= .02
Renal	38 (64.4)	6 (21.5%)	< .001
Hepatic	32 (54.3)	8 (28.5%)	= .02
Neurologic	37 (62.7)	18 (6.3%)	NS
Coagulation	37 (62.7)	11 (3.3%)	= .04

Comparison of organ failure according to SOFA score (Sequential Organ Failure Assessment Score) between IAH and non-IAH patients on admission and during the entire ICU stay. IAH, intra-abdominal hypertension; ICU, intensive care unit; NS, not significant. Values are *n *(percent).

### General characteristics of patients with IAH (Table 8, Figure 3)

**Figure 3:**
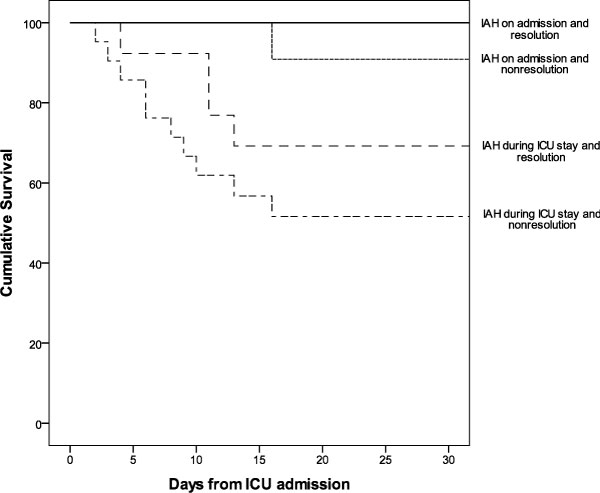
**Kaplan-Meier cumulative survival curve for different subgroups of patients with IAH**. Kaplan-Meier cumulative survival curve for different subgroups of patients with IAH: IAH on admission or during the ICU stay depending on resolution or non-resolution of IAH. IAH, intra-abdominal hypertension; ICU, intensive care unit.

Non-survivors had significantly higher severity scores, number of organ failures, and CRF for IAH, both on admission and during the ICU stay. The more commonly found subtypes of IAH were subacute (51 patients, 86.4%) and grade II (34 patients, 57.6%). IAH was resolved in a high proportion of patients (42.4%) and none presented ACS or required surgical abdominal decompression. Patients in whom IAH was not resolved (57.6%) had a significantly higher mortality rate than patients whose condition was resolved (64.7 vs. 35.2%, *p *= 0.001). Kaplan-Meier survival analysis for patients with IAH on admission or during the ICU stay depending on resolution is shown in Figure 3.

**Table 8:** Characteristics of patients with intra-abdominal hypertension and comparisons between survivors and non-survivors

	Intra-abdominal hypertension	Survivors	Non-survivors	*p *value
Patients (*n*, percent)	59 (100)	32 (54.2)	27 (45.8)	-
Age (years)	59.3 ± 16.4	55.7 ± 16.6	63.5 ± 15.5	NS
Male gender (*n*, percent)	44 (74.6)	23 (71.8%)	21 (77.8)	NS
Body mass index (kilogram per square meter)	27.7 ± 5.6	27.8 ± 6.8	27.6 ± 3.9	NS
APACHE II score, points	24.4 ± 7.8	22.5 ± 8.8	26.6 ± 5.7	.03
SOFA score on day 1, points	8.8 ± 4	7.5 ± 3.7	10.2 ± 3.9	.009
Number of organ failures on day 1, points	1.7 ± 1.1	1.3 ± 1	2.2 ± 0.9	< .001
SOFA score maximum, points	11.7 ± 4.7	10.2 ± 4.2	13.5 ± 4.8	.006
Number of organ failures maximum	2.6 ± 1.3	2.3 ± 1.2	3.1 ± 1.3	.02
Number of categorized risk factors	2.5 ± 0.9	2.3 ± 0.8	2.8 ± 0.9	03
At admission	2.9 ± 0.8	2.7 ± 0.8	3.3 ± 0.7	.003
During ICU stay	5 (8.5)	3 (9.4)	2 (7.4)	-
Classification of IAH according to				
Presentation or development of symptoms (*n*, percent)				
Acute	51 (86.4)	26 (81.2)	25 (92.6)	-
Subacute	3 (5.1)	3 (9.4)	0 (0)	NS
Chronic	6 (10.2)	3 (9.4)	3 (11.1)	NS
Grade of IAP reached during ICU				
stay (*n*, percent)				
I (12-15 mmHg)	34 (57.6)	19 (59.4)	15 (55.5)	NS
II (16-20 mmHg)	14 (23.7)	8 (25)	6 (22.2)	NS
III (21-25 mmHg)	5 (8.5)	2 (6.2)	3 (11.1)	NS
IV (> 25 mmHg)	13 ± 1	14 ± 2	13 ± 1	NS
IAP mean during ICU stay	73 ± 12	74 ± 12	71 ± 12	.001
APP mean during ICU stay	35 (59.3)	21 (65.6)	14 (51.8)	NS
Time of IAH development (*n*, percent)				
On admission	24 (40.7)	11 (34.4)	13 (48.1)	NS
During ICU stay	1 (1-3)	1 (1-3)	1 (1-4)	NS
Day of IAH development during ICU stay^a^	8 (4-16)	7 (4-16)	9 (6-16)	NS
Days with maintained IAH during ICU stay^a^	34 (57.6)	12 (37.5)	22 (81.5)	
Non-resolved IAH (*n*, percent)				
Positive cumulative fluid balance at IAH diagnosis	49 (83)	27 (84.4)	22 (81.5)	
Positive cumulative fluid balance during ICU stay	25 (42.4)	10 (31.2)	15 (55.5)	
ICU stay (days)^a^	1 (7-29)	12 (7-26)	12 (6-33)	
Hospital stay after ICU (days)^a^	29 (13-61)	34 (23-58)	16 (8-65)	

^a^Data are presented as median and interquartile range. APACHE II, Acute Physiology and Chronic Health Evaluation; APP, abdominal perfusion pressure; IAH, intra-abdominal hypertension; IAP, intra-abdominal pressure (mmHg); ICU, intensive care unit; NS, not significant; SOFA, Sequential Organ Failure Assessment. Data are presented as mean ± standard deviation, unless specified.

### Multivariate analysis: predictive and prognostic models (Tables 9, 10 and 11)

#### Predictive model for IAH development in the high risk group (87 patients with ≥2 CRF)

SOFA on admission, obesity, and number of CRF during ICU stay (odds ratio (OR), 2.31; 95% confidence interval (CI), 1.09 - 4.88) were independent predictors of IAH development.

**Table 9:** Logistic regression model for predictors of development of IAH in the high risk group

	Coefficients	Odds ratio	95% confidence interval	*p *value
Age ≥65 years	0.780	2.18	0.66-7.15	.19
Body mass index ≥30, kg/m^2^	2.312	10.09	1.97-51.58	.005
SOFA score at admission	0.153	1.16	1.00-1.35	.04
Number of categorized risk factors for IAH during ICU stay	0.840	2.31	1.09-4.88	.02
Constant	-3.408			

Predictors of development of IAH in the group with ≥2 categorized risk factors for IAH (87 patients). CI, confidence interval; SOFA, Sequential Organ Failure Assessment; IAH, intra-abdominal hypertension; ICU, intensive care unit. Area under receiver operating characteristic curve = 0.80; Hosmer-Lemeshow goodness-of-fit (x^2 ^= 4.22; *p *= 0.835).

**Table 10:** Logistic regression model for predictors of mortality in the high risk group

	Coefficients	Odds ratio	95% Confidence interval	*p *value
Age ≥65 yrs	2.057	3.82	2.08-29.28	.002
APACHE II score ≥20	1.614	5.02	1.11-22.70	.03
SOFA score maximum during ICU stay	0.224	1.25	1.03-1.51	.01
Positive cumulative fluid balance during ICU stay	1.730	5.64	1.50-21.14	.06
No. of categorized risk factors for IAH during ICU stay	1.200	3.32	0.94-11.72	.34
Increased abdominal content during ICU stay	1.007	2.73	0.34-21.78	.08
IAH	1.409	4.09	0.83-20.12	
Constant	-9.908			

Predictors of mortality in the group with ≥2 categorized risk factors for IAH (87 patients). APACHE II, Acute Physiology and Chronic Health Evaluation; CI, confidence interval; IAH, intra-abdominal hypertension; ICU, intensive care unit; SOFA, Sequential Organ Failure Assessment. Area under receiver operating characteristic curve = 0.78; Hosmer-Lemeshow goodness-of-fit (x2 = 3.78; p = 0.870).

**Table 11:** Logistic regression model for predictors of mortality in the IAH group

	Coefficients	Odds ratio	95% confidence interval	*p *value
Age ≥65 yrs	2.811	16.63	2.46-112.30	.004
SOFA score maximum during ICU stay	0.196	1.22	0.97-1.53	.09
No. of categorized risk factors for IAH during ICU stay	2.297	9.94	1.59-62.00	.01
Increased abdominal content during ICU stay	1.847	6.34	0.43-93.31	.17
Non-resolution of IAH	2.577	13.15	22.13-81.92	.006
Constant	-13.953			

Predictors of mortality in the patients with IAH (59 patients). CI, confidence interval; IAH, intra-abdominal hypertension; ICU, intensive care unit; SOFA, Sequential Organ Failure Assessment. Area under receiver operating characteristic curve = 0.80

#### Predictive model of mortality in the high risk group (87 patients with ≥2 CRF)

IAH development was a non-independent predictor of mortality (OR, 4.09; 95% CI, 0.83 - 20.12).

#### Predictive model of mortality in the IAH group (59 patients)

Non-resolution of IAH (OR, 13.15; 95% CI, 22.13 - 81.92), number of CRF during ICU stay (OR, 9.94; 95% CI, 1.59-62.00), and age ≥65 years were independent predictors of mortality. Even though the ROC curve showed good discrimination, the Hosmer-Lemeshow test indicated inadequate calibration across all the probability strata for mortality in the model.

## Discussion

This is a prospective epidemiologic study performed in a population of exclusive critically ill medical patients following the WSACS guidelines for the assessment of IAH (screening, IAP measurement, definitions, and classification recommendations). Other published studies had been performed in mixed populations (medical and surgical), before the WSACS consensus statement, based on different definitions of IAH or limited by incomplete assessment in terms of screening or classification of the IAH [17-21].

Our study revealed several interesting findings. Firstly, there was a large number of patients with high risk (≥2 CRF) for IAH development, and the incidence of IAH in this group was high; in addition, the mortality in this subgroup was significantly higher as compared with the low risk subgroup. Secondly, there was an association of IAH with illness severity and the number/type of RF reported in the literature. Thirdly, IAH was a predictor of mortality in association with other clinical factors. And finally, non-resolution of IAH was an independent predictive factor of mortality. Our results support the importance of implementing screening and assessment protocols, according to WSACS recommendation, in order to identify a subgroup of high risk patients with high mortality and high incidence of IAH whom should be monitored for IAP.

### Screening, risk factors and incidence of IAH

A large number of patients presented ≥2 CRF for IAH development, and the incidence of IAH in this group was high (67.8%) on admission or during the ICU stay. In addition, several high risk patients without IAH might have shown underestimated IAP levels because of the different factors usually present in the critically ill population, such as sedation or gastric/colonic decompression. A high incidence of IAH had also been reported in the literature [17-21]; however, when trying to combine our results with previously described findings, three main methodology issues should be accounted for: a) incidence is recorded on admission or during the first week; b) most reports refer to mixed populations (both medical and surgical); c) definitions of IAH differed across the studies (incidence rates can vary depending on the threshold IAP level used as a diagnostic criterion). Unlike previous studies, our study analyzes the incidence of IAH in a high risk population on admission and throughout the entire ICU stay, following WSACS consensus guidelines, and exclusively in critically ill medical patients. Reintam et al. [17] and Vidal et al. [18] have also used WSACS definitions for IAH, and they report incidence rates of 37% in a mixed population, and 43% in the medical subgroup of a mixed population, respectively.

Numerous conditions predispose to IAH development, and our results are consistent with those from other studies [20,21]. In the high risk group, we found an association between IAH and the CRF proposed by the WSACS, specially the increased abdominal content. Obesity and the number of CRF for IAH during ICU stay were independent predictors of IAH development. Data on fluid balance were only qualitative (positive or negative), and the association between IAH and massive fluid loading in the presence of sepsis and/or capillary leak described in the literature could not be studied.

### Severity scores and organ failure in patients with IAH

Increases in IAP have deleterious effects on end-organ system function by compromising perfusion, thus leading to multiorganic dysfunction (MOD) and poor outcome. In our population, IAH was significantly associated with higher severity of illness scores and incidence of organ failure. In addition, the study design allowed us to compare organ impairment during ICU stay between patients with and without IAH (Table 5). Previous studies analyzed organ impairment only during the first days of ICU stay, thus making it difficult to study the association between MOD and IAH throughout the entire ICU stay.

### Characteristics of IAH

WSACS gives special attention to the IAH classification; this correlates with the patient's profile, namely, whether the patient is medical or surgical [17-21,32-34]. Classification of IAH as acute and with high IAP grades (III-IV) is typical for surgical patients, whereas classification of IAH as subacute and with low-moderate IAP grades (I-II) is characteristic for medical patients [17-21,35]. Our results in medical patients are consistent with this observation. The IAH classification varies depending on the study; however, no studies include all the classification types currently recommended by WSACS. In summary, a complete classification is important for planning future studies and comparing results, and the differentiation between medical or surgical patients is, in our opinion, necessary for a correct analysis based on the differences with regard to the causes and evolution of IAH in those two types of patient's profile.

Fourteen patients reached grade III of IAH, and five grade IV, but none of them developed ACS because a sustained elevation of IAP > 20 mmHg associated with organ deterioration was not detected. In concordance with this, no patient required surgical abdominal decompression.

### Prognostic implications of IAH

Development of IAH has been described as an independent predictor of mortality in mixed populations [20]. In our study IAH was a non-independent predictor of mortality, and this supports the fact that IAH was a marker of mortality in association with other clinical factors. In accordance with this, patients with IAH had higher severity scores and number of CRF for IAH that can independently increase mortality risk. In fact, the number of CRF during ICU stay was an independent predictor of mortality in the group with IAH.

We found a high rate of resolution of IAH in our medical patients, but non-resolution was an independent predictor of mortality, resulting in a considerably high OR (non-resolution meant that death was 13 times more likely). Treatment of IAH was nonsurgical in all cases and none developed ACS, although no conclusions can be drawn in the absence of clinical intervention data. Management guidelines for IAH have been proposed by the WSACS [24] although they are based on studies that analyze therapeutic strategies in isolation and in different patient profiles [36-40].

### Limitations of the study

IAP was not measured in all cases (only in those with ≥2 CRF), and patients with < 2 CRF and no IAP recorded might have developed IAH during their ICU stay (e.g., patients with mechanical ventilation and therefore, diminished abdominal wall compliance but with no RF in other categories and no IAP measured). Therefore, the study followed the WSACS screening recommendations but was not designed to validate them in order to identify a subgroup of high risk patients for IAH development, as compared with a low risk subgroup. The study was underpowered to analyze each RF of the four categories as predictors of IAH (small sample size) or the IAP level as a predictor of mortality (the IAP_mean _in the patients with IAH were relatively low to perform comparisons in the outcome). Not all patients who had their IAP measured were sedated, which could have caused a falsely elevated IAP. Although resolution of IAH was a prognostic factor, our study was not designed to focus on therapeutic management of IAH, and no conclusions can be drawn or ascertainments can be made about the strategies that were effective.

## Conclusions

Critically ill medical patients admitted to the ICU with high risk of IAH development (≥2 CRF) have high morbidity, mortality, and incidence of IAH, so IAP should be measured as recommended by the WSACS. In this subgroup of patients, the presence of IAH is associated with mortality as a non-independent risk factor, and non-resolution of this process is an independent predictor of mortality. Our study supports the implementation of protocols based on WSACS guidelines for IAH screening and assessment to allow an early approach to this syndrome. Large, multicenter randomized controlled clinical trials are needed to confirm our results and to further promote the WSACS recommendations regarding IAH screening and management.

## Abbreviations

APACHE II: Acute Physiology and Chronic Health Evaluation; APP: abdominal perfusion pressure; BMI: body mass index; CRF: categorized risk factors; IAH: intra-abdominal hypertension; IAP: intra-abdominal pressure; ICU: intensive care unit; MOD: multiple organ dysfunction; RF: risk factors; SOFA: Sequential Organ Failure Assessment; WSACS: World Society of Abdominal Compartment Syndrome.

## Consent

Written informed consent was obtained from the patients or their relatives for publication of this case report. A copy of the written consent is available for review by the Editor-in-Chief of this journal.

## Competing interests

The authors declare that they have no competing interests.

## Authors' contributions

All authors read and approved the final manuscript. PS conceived the study and participated in its design and coordination. PS and JM performed the statistical analysis. LA and PG participated in analysis and interpretation of data. IM, MZ and MT collected data. All authors contributed to the writing and preparation of the manuscript.

## Authors' information

PS, MD, PhD; JM, MD, Associate Professor of Medicine, Universidad Complutense de Madrid (UCM), Spain; MT, RN, Chief of Critical Care Nurse Department; IM, MD; MZ, MD; LA, MD, PhD, Chief of Internal Medicine Department, Professor of Medicine, UCM, Spain; PG, MD.
